# Examining health literacy and self-efficacy levels and their association with preventive behaviors of urinary tract infection in Iranian pregnant women: across sectional study

**DOI:** 10.1186/s12905-023-02359-3

**Published:** 2023-05-12

**Authors:** Vajieh Eslami, Seyedeh Belin Tavakoly Sany, Hadi Tehrani, Vahid Ghavami, Nooshin Peyman

**Affiliations:** 1 Department of Health Education and Health Promotion, Faculty of Health, Mashhad University of Medical Sciences, Mashhad, Iran; 2 Department of Health, Safety and Environment, Faculty of Health, Mashhad University of Medical Sciences, Mashhad, Iran; 3 Department of Health Education and Health Promotion, School of Health, Mashhad University of Medical Sciences, Mashhad, Iran; 4 Social Determinants of Health Research Center, Mashhad University of Medical Sciences, Mashhad, Iran; 5 Department of Epidemiology and Biostatistics, Faculty of Health sciences, Mashhad University of Medical Sciences, Mashhad, Iran

**Keywords:** Health literacy, Self-efficacy, Health-promotion, Urinary tract infection, Preventive behaviors, Women health, Health education, Public health

## Abstract

**Objective:**

Urinary tract infection (UTI) is assumed to be associated with the risk of fetal and maternal mortality and morbidity during pregnancy. The potential effect of health literacy and self-efficacy on UTI preventive behaviors among pregnant women has not yet been fully studied. Our objectives were to determine the level of health literacy, self-efficacy, and UTI prevention behaviors in pregnant women, and whether health literacy and self-efficacy are associated with UTI prevention behaviors in pregnant women.

**Design:**

A cross-sectional study was conducted, from November 2020 to December 2020, through a multi-stage sampling design on 235 pregnant women aged between 18 and 42 years in Mashhad, Iran. Data were collected through valid and reliable questionnaires including the Test of Functional Health Literacy in Adults (TOFHLA), General Self-Efficacy Questionnaire (GSE), and research made-preventive behaviors recommendations for UTI disease.

**Results:**

The level of UTI prevention behaviors scores is moderate (71.39 ± 8.58) among women during their pregnancy. Insufficient health literacy and self-efficacy were observed in 53.6% and 59.3% of participants, respectively. The regression model highlighted that 21.20% of the total variance of UTI preventive behaviors was predicted by sociodemographic characteristics, while 40.81% of the variance of UTI preventive behaviors was predicted by health literacy and self-efficacy.

**Conclusion:**

It has been observed that health literacy and self-efficacy are main determinants to improve UTI preventive behaviors. Focusing on an intervention based on health literacy skills may be a practical strategy to promote a healthy lifestyle in this population.

**Supplementary Information:**

The online version contains supplementary material available at 10.1186/s12905-023-02359-3.

## Introduction

Pregnancy is a unique and natural physiological process in women’s lives. However, pregnancy leads to unwanted complications and hormonal changes in the female body that enhance the possibility of urinary tract infection (UTI) [1, 2]. This infection is the most common risk factor during pregnancy which constitutes about 6% of medical referrals. It is estimated that 25% of women get urinary tract infections at least once in their lifetime [3] [4]. The prevalence rate of UTI in pregnant women is 7.2–10.4%, and approximately 20–40% of pregnant women with UTI will develop symptomatic UTI if left untreated [5]. There is evidence that UTI is the main cause of adverse maternal outcomes and obstetrical hospitalization during pregnancy. Likewise, UTI is associated with increasing fetal and maternal mortality and morbidity. Complications of preterm birth may have a life-long impact on both mother and baby. For instance, premature babies often have lung problems, diabetes, heart disease, mental retardation, hearing loss, vision impairment, learning disabilities, and cerebral palsy [3]. Urinary tract infection also causes postpartum endometritis, sepsis, and ultimately maternal shock [4].

Health behaviors during the pregnancy impact pregnancy outcomes [6, 7]. One way to improve women’s health behaviors during pregnancy is to promote their self-efficacy abilities. Self-efficacy abilities are the “individual’s confidence in her or his ability to cope with specific situations“ [8, 9]. Bandura identified four major sources of self-efficacy including previous successful experiences, succession experiences, verbal encouragement, and emotional arousal” [10, 11]. Studies also show that self-efficacy is an important prerequisite for the initiation, adoption, and maintenance of health behaviors because the sense of personal efficacy can lead to differences in the way people think, feel, and act [12]. Therefore, the sense of self-efficacy plays a role in how individuals manage actual situations. There are indications that the level of self-efficacy and high-risk behaviors are negatively interrelated [10, 12, 13].

Health literacy skill is the main driver to improving health outcomes and quality of life [14]. Health literacy has gained critical importance in public health to better manage social and environmental factors improving individual’s health status [15, 16]. The health literacy concept is expanding and now includes information-seeking, critical thinking, communication, problem-solving, decision-making, and personal, social, and cognitive skills to promote the public health system. [17, 18]. Therefore, in addition to the recent body of literature, limited health illiteracy is correlated with high morbidity during pregnancy because mothers with inadequate health literacy are less likely to involve in self-care behaviors and health-promoting behaviors to use preventive healthcare services or to participate in screening tests [17, 19].

Adequate mothers’ ability to understand, evaluate, and use health information is essential during pregnancy, particularly regarding risky health behavior and preventive behaviors [8, 13, 17]. However, despite the growing recognition of the crucial role of health literacy in individuals and community settings, there are limited studies on the health literacy level in pregnant women and its association with health behavior and health outcomes during pregnancy [17]. In addition, the exact nature of functional skills to improve UTI preventive behaviors is still unclear.

In recent years, limited studies examined the association between self-efficacy, health literacy level, and health outcomes during pregnancy [13, 17]. There has not yet been any comprehensive study on the association of health literacy and self-efficacy with UTI- prevention behaviors in pregnant women [6, 17]. Previous studies have mostly focused on lifestyle factors and diagnostic methods during pregnancy. The reasons why pregnant women adhere or do not adhere to preventative behaviors for UTI may lie in other factors beyond women’s s awareness, beliefs, and perceptions [3, 4]. Several studies showed that although women have appropriate awareness in the field of UTI prevention, the prevalence of infections in pregnant women is increasing [3, 4, 20] because women’s confidence and skills are not enough to perform appropriate behaviors [3, 21, 22]. Therefore, it seems that raising awareness alone cannot lead to the prevention of UTI.

Women experience intense fears and conflict related to adoption with the first pregnancy. Individuals with appropriate social support, self-efficacy, and health literacy may have acceptable medical and psychological adaptation to pregnancy. There are few studies conducted on women’s experiences of their first pregnancy from a caring perspective and woman’s lifeworld. Likewise, women in early pregnancy need more midwifery care in comparison with other pregnant women. Taking women’s medical and psychological adaptation in the first pregnancy may prevent future suffering during childbirth.

Therefore, given the high prevalence of UTI during pregnancy and their adverse effect on women’s health, this study aims to determine the level of health literacy, self-efficacy, and UTI preventive behaviors among women in early pregnancy, and whether health literacy and self-efficacy are associated with UTI prevention behaviors in pregnant women.

## Materials and methods

### Sample Population and Study Procedures

We conducted this cross-sectional study on 235 pregnant women in Mashhad, Iran, from November 2020 to December 2020. Pregnant women included in this study if they (a) were satisfied to participate; (b) were in the first pregnancy; (c) did not have UTI; (f) able to complete all questionnaires; (g) did not hospitalize in the past three months, and were in gestational age from 12 to 18 weeks. Participants were also excluded if they (a) were unable to give informed consent; (b) had suffered diabetes, kidney disease, and high blood pressure; (c) did not fully answer the questionnaires.

All eligible women filled out the consent form and completed the research tool in a written format. The sample size was calculated using the following formula:

$$n = \frac{{z_1^2 - \alpha /2^{\sigma ^2}}}{{{d^2}}}$$

Where n is the sample size, α is the first type, Z is the table-based normal distribution index that is considered at 5% type-one error (P < 0.05), σ represents the small variable variance, and d shows the accuracy of quantitative variable estimation. In this study, a first type error, z, σ, and d equal to 0.05, 96.3, 7.38, and 0.99, respectively. After adjusting for the non-response of 10%, 238 women were considered as the sample size, and 235 pregnant women were included in the data analysis [23].

We used a multi-stage sampling method to designate the study locations. We divided the city into 6 regions (or clusters) based on the municipal parts. In first stage, four regions (main clusters in this study) were purposely selected based on the number of households and socioeconomic diversity. The second-stage sampling consists of a simple random sample to select two health centers from the main clusters (total 8 health centers). Subsequently, we used available sampling to select eligible participants from the health centers. A total of 523 pregnant women were included, of which 294 did not meet the inclusion criteria, and 3 pregnant women did not answer all the items in the questionnaire. Finally, 235 women were included in the data analysis and completed all questionnaires.

### Measures

In each interview, we informed all eligible participants about the purpose of the study, and they completed informed consent. Next, we asked all women about their demographic characteristics (age, employment status, income status, education level, and weight and height). All clinical information was extracted from electronic health records (EHRs). We used a questionnaire on UTI preventive behaviors, TOFLHA, and Scherer self-efficacy to measure UTI preventive behaviors, health literacy, and general self-efficacy.

#### General Self-Efficacy Questionnaire (GSE)

This questionnaire is a Likert format 17-item to rate the strength of the individual’s confidence when they engage in new situations despite various difficulties [24]. This scale measures three aspects of behavior, including the desire to initiate behavioral, resistance to obstacles, and trying to complete the task (e.g., “I am a self-reliant person “, “I avoid facing difficulties”, and “When I make plans, I am certain I can make them work”). Items in this construct ranged from 1 (no confidence) to 5 (great confidence). The maximum score a person could achieve on this scale is 85, with a minimum score of 17(Table S1). Items in this construct ranged from low (17–40), moderate (41–62), and appropriate (63–85). Previous studies in Iran found internal consistency reliabilities of GSE to be moderate to high (Cronbach’s alpha = 0.74 to 0.86) [25].

#### Test of Functional Health literacy in adults (TOFHLA)

Health literacy was evalauted based on an individual’s ability to read, understand, and act on health-context materials. We used TOFHLA to examine health literacy status [26] becuase it is a valid and reliable measure to evaluate the literacy skills in diffrent populations. Likewise, TOFHLA represents health-related duties and its association with fluid cognitive abilities [8, 26]. This questionnaire consists of 17 items of numerical ability and 50-item of reading comprehension tests, which takes 22 minutes. The numerical section measures a person’s ability to understand and act based on the recommendations given to him by physicians and health educators who need calculations. The reading comprehension section evaluates participants’ ability to read and understand the three texts under the headings of preparation for upper gastrointestinal imaging, patient rights, and responsibilities in insurance policy forms. All participants signed standard hospital consent forms. All participants signed standard hospital consent forms. Items were rated from inadequate (0–59), marginal (60–74), and adequate (75–100) [24]. The reliability of this tool was confirmed for the numerical ability (alpha = 0.79) and the reading section (alpha = 0.88). Content validity ratio (CVR) and content validity index (CVI) were 0.78 and 0.79, respectively [27].

#### Questionnaire to test UTI preventive behaviors

This is a researcher-made questionnaire that was designed based on quantitative and qualitative databases related to the prevention and control of UTI in pregnant women [28]. During the preparation of the questionnaire, the necessary consultations were obtained from ten specialists and professors in midwifery, gynecology, health education and promotion, and urology (panel of experts in this study). This questionnaire consists of 25 items to measure preventive behaviors of UTI with focus on health behaviors related to clothing (4 items), nutrition (6 items), urination (2 items), sexual habits (6 items), and an individual’s health behaviors (7 items). All items in this questionnaire are in a Likert format from the range of never (0) to always (4). All scores are calculated on a scale of 100 and items in these constructs ranged from poor (0–50), moderate (51–75), and acceptable (76–100). The questionnaire was given to an expert panel to review the necessity and relevance of all questions to quantify the content validity ratio (CVR) and content validity index (CVI), which were equal to 0.84 and 0.94, respectively. It was an acceptable level in the present study. We also used the internal consistency method to measure the reliability and coefficients of the questionnaire. Chronbach’s alpha showed that all the coefficients were satisfactory and more than 0.72. Piloting is conducted to test survey questionnaire before beginning the study to understand how questionnaire works in the field. Piloting helps us to detect obstacles, remove difficulties, and obtain appropriate and accurate data. The questionnaire was distributed twice and with an interval of two weeks among 25 pregnant mothers (10% of study sample size) whose demographic characteristics were like the studied population.

### Statistical analysis

The SPSS software version 18 (Chicago, Illinois) was used in this study to conduct a series of descriptive and bivariate statistics tests. We used descriptive analyses (mean score, standard deviation, and frequency) to measure socio-demographic characteristics and the level of health literacy, self-efficacy, and UTI prevention behavior. Bivariate tests (t-tests, Man-Whitney, Kruskal Wallis, and fisher’s test) was used to determine group differences between qualitative variables (education, employment status, family income, vomiting, and history of pre-pregnancy). Correlations among UTI prevention behaviors, health literacy, self-efficacy, and quantitative variables were calculated using Spearman and Pearson’s correlations. In order to examine whether preventive behaviors of UTI are empirically associated with health literacy, self-efficacy, and demographic variables, a hierarchical multiple regression analysis was used, and all variables were entered in this model based on logical and theoretical considerations in this study to compute the contributions of the independent variables. A p-value lower than 0.05 shows statistical differences occurred between the different groups.

## Results

### Descriptive statistics

The result showed that 74.4% of the eligible women were housewives, and around half of them were from moderate-income families (52.1%) with high school diplomas or below (57.1%) (Table 1).

The total score for health literacy was in the range of inadequate literacy level, and 53.6% of participants showed inadequate health literacy levels (Table 1). The self-efficacy was 47.62 ± 14.52, suggesting that self-efficacy status is in the range of moderate level in this study’s participants. Likewise, 81.92% of the participants had low or moderate self-efficacy scores during their pregnancy. The UTI prevention behaviors among participants were moderate (71.39 ± 8.58). The results of the preventive behaviors of UTI showed that most women had difficulty in health behaviors related to clothing (69.51 ± 11.55), nutrition (67.26 ± 11.31), and urination (70.74 ± 18.36) (Table 1 and Table 2). Approximately 30–65% of women did not have healthy behaviors and used tight pants with inappropriate material, drank soft drinks, tea, coffee, and sour drinks, and delayed voiding.

**Table 1:** Subject characteristics and prevention behaviors

Variables characteristics *(n = 238)*		
Age, *years, mean ± SD*	Range: 18–42	26.77 ± 5.281
Gestational age, *(weeks), mean ± SD*	Range: 6–31	20.75 ± 6.948
Weight, *(kg), mean ± SD*	Range: 45–105	72.121 ± 12.09
Height, *(cm), mean ± SD*	Range:135–175	163.44 ± 6.231
BMI^d^, *kg/m*^*2*^, *mean ± SD*	Range: 18.07–39.23	26.95 ± 3.99
Women’s Education level, *n (%)*	Diploma/under diploma	136(57.1)
	Higher education	102(42.9)
Women’s Employment Status, *n (%)*	Housewives	177(74.4)
	Employed	61(25.6)
Spouse’s education level, *n (%)*	Diploma/under diploma	140(58.8)
	Higher education	98(41.2)
Spouse’s Employment Status, *n (%)*	Employee	63(26.5)
	Freelance	110(46.2)
	Worker	50(21.0)
	Unemployed	15(6.3)
Family Income, *n (%)*	Low	114(47.9)
	Moderate	124(52.1)
Vomiting in pregnancy, *n (%)*	Yes	117(49.2)
	No	121(50.8)
History of pre-pregnancy UTI, *n (%)*	Yes	68(28.6)
	No	170(71.4)
Total Health literacy, *mean ± SD*	Range: 30.24–89.45	56.10 ± 16.23
Health literacy skills, n (%)	Inadequate:	128 (53.6)
	Marginal:	71(30)
	Adequate:	39 (16.4)
Total self-efficacy, mean ± SD	Range: 25–81	47.62 ± 14.52
Self-efficacy ability, n (%)	Low	96(40.33)
	Moderate	99(41.6)
	Appropriate	43(18.06)
Total preventive behaviors, mean ± SD	Range: 50–94	71.39 ± 8.58
Clothing habits, *mean ± SD*	Range: 37.5–100	69.51 ± 11.55
Nutrition, *mean ± SD*	Range: 41.67–95.83	67.26 ± 11.31
Urination, *mean ± SD*	Range: 25–100	70.74 ± 18.36
Health behaviors, *mean ± SD*	Range: 46.43–100	74.17 ± 13.03
Sexual behavior, *mean ± SD*	Range: 25–100	73.87 ± 14.63

±: Showing mean score (standard deviation); n: number of eligible participants; ^**d**^ Body Mass Index **(**BMI) was classified as underweight (< 18.5 kg/m^2^), normal (18.5–24.9), overweight (25–29.9) and obese (> 30); %: percent of total participants

**Table 2:** Distribution of urinary tract infection prevention behaviors

Questions (n = 238) *n (%)*	Always	Sometime	Rarely	Never
**Clothing habits**				
I wear loose pants	78(32.8)	79(33.2)	54(22.7)	27(11.3)
I wear cotton underwear	88(37)	96(40.3)	48(20.2)	6(2.5)
I change my underwear 3 or more times a week	119(50)	66(27.7)	50(21)	3(1.3)
I use underwear	119(50)	51(21.4)	49(20.6)	19(8)
**Nutrition**				
I drink 8 glasses or more of water daily	67(28.2)	66(27.7)	76(31.9)	26(12.2)
I drink 3 or more cups of tea a day	68(28.6)	65(27.3)	74(31.1)	31(13)
I drink 1 glass or more of soft drink a day	14(5.9)	61(25.6)	102(42.9)	61(25.6)
I drink 1 cup or more of coffee a day	13(5.5)	42(17.6)	61(25.6)	122(51.3)
I use yogurt and milk daily or one day.	69(29)	91(38.2)	63(26.5)	15(6.3)
1 to 2 times a week I use sour drinks such as (barberry juice or blueberry, etc.)	25(10.5)	57(23.9)	80(33.6)	76(31.9)
**Urination**				
When I feel like urinating, I refrain from urinating	30(12.6)	58(24.4)	68(28.6)	82(34.5)
I urinate about 1 h after the first feeling of urination	65(27.3)	91(38.2)	54(22.7)	28(11.8)
**Health behaviors**				
After using the toilet, I first clean the urethra and then the anus	116(48.9)	51(21.5)	43(18.1)	27(11.4)
I use the pitcher to purify myself	23(9.7)	22(9.2)	54(22.7)	139(58.4)
After using the toilet, I use a towel to dry myself	94(39.5)	55(23.1)	51(21.4)	38(16)
I use paper towels when using public toilets	83(34.9)	59(24.8)	45(18.9)	51(21.4)
I take a standing bath	110(46.2)	73(30.7)	44(18.5)	11(4.6)
I take a bath 3 or more times a week	86(36.1)	77(32.4)	65(27.3)	10(4.2)
I dry my underwear in the sun	48(20.2)	82(34.5)	71(29.8)	37(15.5)
**Sexual behavior**				
I urinate before sex	65(27.3)	82(34.5)	65(27.3)	26(10.9)
I wash my genital area before sex	69(29)	90(37.8)	58(24.4)	21(8.8)
I urinate shortly after sex	89(37.4)	95(39.9)	47(19.7)	7(2.9)
I wash my genitals shortly after sex	103(43.3)	70(33.6)	45(18.9)	10(4.2)
My wife washes the genital area before intercourse	65(27.3)	86(36.1)	60(25.2)	27(11.3)
If I have a urinary tract infection, I will not approach for 2 weeks	84(35.3)	92(38.7)	42(17.6)	20(11.3)

n: number of eligible participants; %: percent of total participants

### Biovariate analyses

Based on our results, women with higher education and income significantly (p < 0.05) showed better self-efficacy, health literacy, and prevention behaviors scores than women who have lower levels of education and income (Table 3). Similarly, women’s age (p = 0.001) and spouse’s education (p = 0.001) were positively and significantly (p < 0.05) associated with women’s preventive behaviors toward UTI (Table 3 and Table 4).

Health literacy was positively and significantly correlated with self-efficacy and preventive behaviors of UTI. There is also a statistically significant positive correlation between self-efficacy and preventive behaviors of UTI (P < 0.001; r = 0.998). In addition, all the aspects of UTI prevention behaviors are significantly associated with self-efficacy and health literacy (Table 4). The information in Table 4 showed that the correlation between preventive behaviors of UTI, health literacy, and self-efficacy was higher than other outcomes.

**Table 3:** Relationship between qualitative variables and UTI prevention behaviors, self-efficacy and health literacy

Variables *(n = 238)* *mean ± SD*		Self-efficacy	Health literacy	Preventive behaviors
Women’s Education level	Diploma/under diploma	44.97 ± 14.14	57.88 ± 11.47	69.90 ± 8.54
	Higher education	51.15 ± 14.33	54.30 ± 12.33	73.38 ± 8.27
	p-value	*p*=0.002	^2^*p*=0.075	^1^*p*=0.001
Women’s Employment Status	Housewife	47.06 ± 15.51	56.11 ± 8.68	71.11 ± 8.68
	Employed	49.26 ± 14.56	57.19 ± 8.32	72.19 ± 8.32
	p-value	*p*=0.399	*p*=0.172	*p*=0.408
Spouse’s education level	Diploma/under diploma	44.75 ± 14.12	57.35 ± 12.85	69.74 ± 8.48
	Higher education	51.72 ± 14.16	54.91 ± 10.03	73.75 ± 8.21
	p-value	*p*=0.001	*p*=0.229	*p*=0.001
Spouse’s Employment Status	Employee	53.20 ± 13.58	52.35 ± 12.57	74.57 ± 7.68
	Freelance	47.76 ± 14.53	55.12 ± 12.77	71.51 ± 8.49
	worker	49.48 ± 13.89	56.02 ± 10.97	68.32 ± 8.76
	Unemployed	46.33 ± 11.99	60.9 ± 12.66	67.40 ± 7.75
	p-value	*p*=0.061	*p*=0.347	3*p*=0.035
Family Income	low	43.74 ± 13.98	57.08 ± 14.98	69.12 ± 8.58
	Moderate	51.19 ± 14.15	55.67 ± 15.73	73.48 ± 8.09
	p-value	*p*=0.001	*p*=0.481	*p*=0.001
Vomiting in pregnancy	Yes	46.55 ± 14.95	56.98 ± 15.90	70.64 ± 9.06
	No	48.33 ± 14.96	55.75 ± 14.87	72.11 ± 8.07
	p-value	*p*=0.189	*p*=0.541	*p*=0. 16
History of pre-pregnancy UTI	Yes	48.33 ± 14.96	55.35 ± 17.34	71.75 ± 9.63
	No	47.34 ± 13.94	56.75 ± 14.53	71.25 ± 8.16
	p-value	*p*=0.668	*p*=0.558	*p*=0.897

±: Showing mean score (standard deviation); n: number of eligible participants; ^1^Man Whitney ^2^T test ^3^Kruskal Wallis ^4^Fisher’s exact test, P-value shows significant differences between main variables, self-efficacy, and health literacy; %: percent of total participants.

**Table 4:** **Relationship of quantitative variables and UTI prevention behaviors, self-efficacy and health literacy**

Variables*(n = 238)*	Health literacy		Self-efficacy		Preventive behaviors	
	R	p-value	R	p-value	R	p-value
Age	-0.057	0.385	0.203	0.002	0.230	0.001
Gestational age	-0.043	0.506	-0.014	0.828	-0.012	0.859
Weight	-0.042	0.524	0.019	0.775	0.070	0.280
Height	-0.012	0.855	-0.095	0.145	-0.098	0.131
BMI	0.068	0.294	0.054	0.403	0.068	0.294
Self-efficacy	0.881	0.001	1	0.001	0.998	0.001
Health literacy	1	-	0.881	0.001	0.959	0.001
Preventive behaviors	0.959	0.001	0.998	0.001	1	-

P-value shows whether the correlation coefficient is significant or not; R-value shows the level of correlation between variables.

### Association analysis

According to the regression model, socio-demographic variables (step 1) accounted for 20.20% of the variance for UTI preventive behaviors. In step 2, adding self-efficacy resulted in a 21.79% increase in the explained variance of UTI preventive behaviors. In step 3, health literacy skills, including numerical ability and reading comprehension skills were also added to Model 3, resulting in a 30% increase in the explained variance of prevention behaviors. The final model showed that 70.61% of the total variance in prevention behaviors was predicted by age, family income, education level, self-efficacy, and health literacy skills. In this study, UTI preventive behaviors are positively correlated with family income, education level, self-efficacy, and health literacy skills, while UTI prevention behaviors are negatively correlated with age (Table 5).

**Table 5:** Summary of Hierarchical Regression analysis for variables predicting UTI prevention Behavior

Determinants *(n = 238)*	Step 1		Step 2		Step 3	
	*Beta*	*t*	*Beta*	*t*	*Beta*	*t*
Constant		3.13***		2.81**		1.7*
Mean Age	-0.17	-2.21*	-0.16	-2.02*	-0.15	-1.81
Gestational age	-0.09	-1.32	-0.06	-1.11	-0.04	-0.80
Women Education Level	0.22	2.36*	0.20	2.32*	0.19	2.23*
Spouse’s education level	0.20	2.26*	0.18	2.20*	0.16	2.01*
Women’s Employment Status	-0.11	-1.40	-0.09	-1.35	-0.06	− 0.1.12
Spouse’s Employment Status	0.07	1.12	0.05	1.02	0.03	72
BMI	-0.12	-1.40	0.09	1.27	0.06	0.95
Family Income	0.20	2.26*	0.17	2.18*	0.15	2.04
History of pre-pregnancy UTI	-0.13	-1.42	-0.12	1.38	-0.09	-1.13
Vomiting in pregnancy	0.03	0.52	0.03	0.51	0.02	0.32
Self-efficacy			0.19	2.52**	0.17	2.37**
Health literacy skills						
Numerical ability					0.29	3.82**
Reading comprehension					0.42	5.61***
R^2^	0.20		0.41		0.71	
R^2^ Changes	0.18		0.28		0.39	
F	3.94		5.75		7.35	
P	< 0.001		< 0.001		< 0.001	

The β values are called regression weights and are computed in a way that minimizes the sum of squared; Dependent Variable: prevention Behavior; *** p < 0.001, ** p < 0.01, * p < 0.05

## Discussion

The primary aim of this study was to investigate the level of health literacy, self-efficacy, and preventive behaviors toward UTI in pregnant women referring to health centers in Mashhad, and to examine the association of health literacy and self-efficacy with preventive behaviors of UTI during pregnancy. The prevalence of UTI in pregnant women is considered one of the most important public health concerns. Thus, it is necessary to improve preventive behaviors toward UTIs during pregnancy due to the complications of the mother and fetus [1, 3].

### Preventive behaviors status

Regarding the first objective of this study, the UTI preventive behaviors among pregnant women were in the range of moderate level, suggesting that pregnant women needed more education in this field. This result is in line with other studies in Iran, which report a moderate level of UTI prevention behaviors [29, 30]. However, there is inconsistency in some reports in Iran [30–33] because a variety of factors impact UTI preventive behaviors in Iranian women such as knowledge [31–33], attitude [30, 32], subjective norm [30, 31], and socio-demographic factors [18, 33]. In the study of Jalali et al., [32] and Ahmadi., et al, [30], mean score of UTI preventive behaviors among Iranian women was in the range of low level because most women did not have adequate knowledge and attitude about the causes of UTI and its complications and adverse effects [30] [32]. Also, another study on UTI in pregnant women showed that physicians, health providers, and husbands were influential in promoting mother motivation to follow UTI prevention behaviors [29, 30, 33].

The results of this study depict associations between some socio-demographic factors and the prevention behavior of UTI. The women’s age and education had a significant positive effect on women’s preventive behaviors, which shows that mothers with a high age and educational attainment had a greater intention to perform UTI preventive behaviors during their pregnancy compared to other pregnant women. These results are consistent with existing empirical studies that report that age [13, 32, 33], family income [17, 31, 33], and education level [30–33] have a significant contribution to disease prevention behavior [22, 33, 34]. On the other hand, Haider et al. reported lower quality of life in mothers with a lower social and economic class, which reduced the rate of prevention behaviors [35]. Therefore, our findings support a significant relationship between UTI preventive behaviors, age and education.

The study revealed that family income, spouses’ education, and employment were statistically effective in improving respondents’ preventive behaviors like the results of several studies [30–33]. Women, whose her husbands had higher education and were employed, were more involved in hygienic practices on UTI prevention during their pregnancy compared with other pregnant women. The reason was likely due to a significant association between the level of education and appropriate knowledge about UTI [22, 33, 34]. Most related studies revealed that education level have a significant association with the appropriate knowledge about UTI, hygienic practices, and prevalence of UTI during pregnancy [22, 33, 34]. Likewise, physicians and women’s husbands were influential in promoting the motivation of pregnant women to follow hygiene practices about UTI [30–33]. In the current study, women who lived in a family with low income showed low UTI prevention behaviour. Our finding also indicated that the low level of hygienic practices among pregnant women was more common in those from poorer households and with unemployed husbands. Therefore, considering the sociodemographic profile is an advantaged characteristic to evaluate community health, especially in the underprivileged population [17]. A study conducted to assess pregnancy-related risk factors among women of low economic status showed that despite the high knowledge and education base, high-risk condition and behaviors existed [30–33]. Knowledge and education alone are not enough to control behavior [17]. Therefore, considering the sociodemographic profile is an suitable characteristic to assess community health, especially in the underprivileged population.

Our finding identified several modifiable eating habits, hygienic practices, and behaviors related to the prevalence of UTI among pregnant women. In this study, approximately 30–65% of women did not have correct behaviors and accurate information about clothing habits, food habits, and urination behaviors during pregnancy (Table 2). The specific results of this study revealed that these women used tight pants with inappropriate material, drank more soft drinks, tea, coffee, and sour drinks, delayed voiding, and had poor healthy behaviors. This result is consistent with the study of Jalali et al. [32] and Ahmadi et al. [30] that indicated most women in their studies did not have enough knowledge regarding clothing way and food habits to prevent UTI. Likewise, several studies indicated that inappropriate food habits are the most comment risk factor for UTI recurrence during pregnancy [17, 32, 33]. A continuous empowerment intervention program based on all aspects of UTI prevention behaviors is essential to maintain the health and to avoid the diseases [29, 30].

### Health literacy and self-efficacy status

Participants in this study showed inadequate health literacy. In 2019, Charghcheian et al. examined the situation of health literacy and self-efficacy status in Iranian women based on a meta-analysis and systematic review [13]. They reported that the overall health literacy and self-efficacy are moderate in Iranian women across 27 studies with 18,075 samples [13]. According to our findings, it seems that the level of health literacy has decreased compared to a study that was conducted by Charghcheian et al., in 2019. This inconsistency in the level of health literacy is more likely related to respondents’ social health determinants such as limited financial resources, poor quality jobs, and low educational attainment [17, 32, 33]. Similarly, other studies have highlighted the considerable effect of economic and social characteristics on inadequate health literacy [17, 18]. They reported that the level of health literacy at the local level is associated with conditions in which individuals are born, live, grow, work, and age [8, 36]. Likewise, this inconsistency in the status of health literacy could be related to the situation of the COVID-19 pandemic. In Iran, pregnant women were regulary receiving primary care from the healthcare centers (health houses) before the COVID-19 pandemic and lockdown [37]. Since telemedicine was limited in Iran because of improper infrastructure and facilities, pregnant women had low access to medical and healthcare centers during the lockdown period [22].

Average self-efficacy score was moderate in this study. However, self-efficacy is low in 40.33% of total participants. All women were recruited from low or moderate-income families, which may lead to the assumption that these participants have lower self-efficacy than the general population of women in Iran. Several studies repeatedly point out that women in situations below the poverty level are more likely to possess inadequate health literacy and self-efficacy [17, 18]. For example, in 2021, Nawabi et al. systematically reviewed overall health literacy among pregnant women [17]. They indicated that women who lived in high-income countries and cities usually had better health literacy during pregnancy [17]. Since health literacy and self-efficacy have been identified as crucial skills to prevent non-communicable diseases and communicable diseases, it is necessary to take immediate action to improve health literacy and self-efficacy skills among different groups of the population, particularly during the COVID-19 pandemic [8, 36].

### Association assessment

Our findings showed the potential effect of self-efficacy on women’s UTI-preventive behaviors during pregnancy. It seems that self-efficacy plays a role in the initiation and adoption of UTI-prevention behaviors among pregnant women. Refat Mohamed et al. [38] and Rahimi et al. [29] investigated the effect of education based on self-efficacy theory to control UTI among pregnant women showed the same results. They reported that self-efficacy had a significant influence on initiating, adopting, and maintaining healthy behavior and act as the main moderator of the association between behaviors, knowledge, and motivation [29, 38, 39]. Our results also show that mothers with a high family income, age, and educational attainment had a greater self-efficacy and attention to perform UTI prevention behaviors during their pregnancy compared to other pregnant women [13, 17, 18]. Since, cross-sectional studies evaluate observations and are unable to examine causative association, we recommend conducting strong evidence-based studies such as a randomized controlled trial to provide subsequent adequately-powered data on such associations.

With regard to association between health literacy and the preventive behaviors against UTI, our finding highlighted that health literacy skills are a stronger determinant that influences UTI-preventive behaviors in pregnant women compared to socio-demographic characteristics and self-efficacy. This could be due to the effective role of health literacy in promoting individual’s attitudes, perceptions, and responsibilities for maintaining their health [8, 40]. It seems that women with adequate health literacy may well understand health information and have suffusion self-efficacy to be actively engaged in their care [18, 41]. Limited studies examined the effect of health literacy on UTI prevention behaviors among pregnant women [8, 41]. Although recent health promotion studies have mostly focused on lifestyle factors and diagnosis methods during pregnancy, a stronger focus needs to be placed on promoting women’s health literacy skills to enhance women’s confidence and ability to promote health prevention behaviors toward the type of diseases [37, 40, 42]. In this case, focusing on health literacy skills can lead to long-term health behaviors and benefits [7, 41, 42]

In this study, a significant strong association was also observed between health literacy and self-efficacy. This result is consistent with recent studies that have investigated the association between self-efficacy and health literacy [13, 19, 29] and their effect on health outcomes and health behaviors [3, 8]. They indicated that if a good relationship is established, health literacy and self-efficacy might positively predict good health care behavior [8, 36, 43]. Without health literacy and self-efficacy skills in performing a specific act like preventive behaviors or self-care action, behavioral capability (knowledge, attitudes, and skills) alone is not able to generate appropriate preventive behavior [13, 18, 29].

### Limitation

This study is subject to the following limitations: the present study was using a self-report tool, which might have led to issues such as recall problems, distortion, over-estimation, or underestimation of scores. However, our researcher confirmed overall authenticity responses, and all responses were closely linked to women’s health characteristics in Iran. Likewise, our findings were obtained from a cross-sectional study, and in a longitudinal study, the causal correlation between them could be better understood. We could not able to examine the stress factor and special mental health during the lockdown. This work was conducted in a metropolis city of Iran that lead to a homogeneous population, and therefore our finding could not be representative for entire communities. Although these biases could not be ignored, we tried to reduce information bias by performing the interviews without rushing and giving the appropriate time and information to the study participant to reply all items.

### Practical implication

Our findings help to better explain the modifiable mechanism (e.g., socioeconomic condition and adequate health literacy) to control and manage UTI and health care delivery during pregnancy even in low-middle income groups. With the study depicting inadequate levels of health literacy in pregnant women, conducting integrated and cross sector interventions to improve health literacy is essential during pregnancy, not only because an adequate health literacy level is critical for the women’s health and unborn child’s health, but also because levels of health literacy affect other behaviors and health outcomes during pregnancy. This type of intervention empowers individuals to be greatly involved in their health care, address the deficiencies to improve patients’ health holistically, and improve their healthy behavior.

## Conclusion

It is the first quantitative data on pregnant women that was investigated UTI prevention behaviors in Iran. The main strengths of our study are examining the mediating role of health literacy and self-efficacy applying a standardized scale among pregnant women (as a vulnerable population) living in a middle-income country. The result of this study indicated that the level of UTI prevention behaviors and self-efficacy among pregnant women were in the range of moderate level. Likewise, inadequate health literacy is still common in pregnant women in Iran. Multiple factors such as age, income, education, health literacy, and self-efficacy may affect UTI prevention behaviors in women during pregnancy. Results highlighted that health literacy skills are a stronger determinant that influences UTI-preventive behaviors in pregnant women compared to socio-demographic characteristics and self-efficacy.

## Electronic supplementary material

Below is the link to the electronic supplementary material.

Supplementary Material 1 General Self-Efficacy Questionnaire

## Data Availability

Datasets used and/or analyzed during the current study are available from the corresponding author on reasonable request.
