# Supplementary material for: Examining health literacy and self-efficacy levels and their association with preventive behaviors of urinary tract infection in Iranian pregnant women: across sectional study
Source: BMC Womens Health. 2023 May 12;23:258. doi: 10.1186/s12905-023-02359-3 (PMC10180610; doi:10.1186/s12905-023-02359-3)
Supplement: Supplementary file 1 — Supplementary Material 1 General Self-Efficacy Questionnaire [file 12905_2023_2359_MOESM1_ESM.docx]

**Supplementary Material:**

**Table S1.** Scherer General Self-Efficacy Questionnaire.

|  | Questions (n= 238) *n (%)* | Completely disagree | Disagree | Average | Agree | Quite agree |
| --- | --- | --- | --- | --- | --- | --- |
| 1 | When I make plans, I am certain I can make them work. | 7(0.9) | 40(16.4) | 37(15.5) | 137(60.9) | 17(6.4) |
| 2 | One of my problem is that I can not get down to work when I should. | 10(2.7) | 103(44.5) | 57(23.6) | 57(23.6) | 15(5.5) |
| 3 | If I can’t do a job the first time, I keep trying until I can. | 15(4.5) | 48(20) | 49(20.9) | 105(46.4) | 21(8.2) |
| 4 | When I set important goals for myself, I rarely achieve them. | 27(10) | 84(36.4) | 45(19.1) | 29(11.8) | 53(22.7) |
| 5 | I give up on things before completing them. | 25(9.1) | 88(38.2) | 47(20) | 69(30) | 9(2.7) |
| 6 | I avoid facing difficulties. | 29(10.9) | 98(42.7) | 31(12.7) | 57(24.5) | 23(9.1) |
| 7 | If something looks too complicated, will not even bother to try it. | 17(5.5) | 117(51.8) | 47(20) | 39(16.4) | 17(6.4) |
| 8 | When I have something unpleasant to do, I stick to it until I finish it. | 38(15.5) | 53(21.8) | 47(20) | 75(32.7) | 25(10) |
| 9 | When I decide to do something new, go right to work on it. | 33(12.7) | 40(16.4) | 61(26.4) | 81(35.5) | 23(9.1) |
| 10 | When trying to learn something new, I soon give up if I am not initially successful. | 25 (9.1) | 116(50.9) | 21(8.2) | 41(17.3) | 35(14.5) |
| 11 | When unexpected problems occur, I don’t handle them well. | 27(10) | 90(39.1) | 57(24.5) | 45(19.1) | 19(7.3) |
| 12 | I avoid trying to learn new things when they look too difficult for me. | 28(10.9) | 94(40.9) | 65(28.2) | 29(11.8) | 21(8.2) |
| 13 | Failure just makes me try harder. | 13(3.6) | 112(24.5) | 29(11.8) | 99(43.6) | 39(16.4) |
| 14 | I feel insecure about my ability to do things. | 26(10) | 94(40.9) | 47(20) | 53(22.7) | 17(6.4) |
| 15 | I am a self-reliant person. | 13(3.6) | 50(20.9) | 47(20) | 95(41.8) | 33(13.6) |
| 16 | I give up easily. | 18(6.4) | 124(54.5) | 61(26.4) | 21(8.2) | 13(4.5) |
| 17 | I do not seem capable of dealing with most problems that come up in life. | 32(12.7) | 102(44.5) | 59(23.6) | 39(16.4) | 9(2.7) |
